# A Relief for Clean Medical Practice

**Published:** 1914-05

**Authors:** G. Henri Bogart

**Affiliations:** Paris, Illinois


For Texas Medical Journal.

### A Relief for Clean Medical Practice.

BY G. HENRI BOGART, M. D., PARIS, ILLINOIS.

The faker, the quack, and the commercial medical pirate have not only served to bring into disrepute the high, honorable practice of the profession, but they are the cause of terribly incalculable evil to the general public.

The heartless beast who advertises a certain cure for tuberculosis, not alone rouses false hopes, but he leads his trusting victims to trust his sword of lath, in the battle with the grim destroyer, until it is too late for science to help; he murders those who trust his siren plea, for the paltry sum paid for his useless remedy.

The other slimy, filthy beast plays on the fears of the youthful, at the stormy period when they are sailing life's bark into the all-too-frequently unchartered seas of adolescence, and leads many onto the rocks of hypochrondria and distorted wreckage of the pure passion of sexuality, perverted for all time, or he appeals to those who have suffered from the pitfalls of ignorance in the social evils, with its damnable lie of sex necessity, as so simply painted in the story of "Damaged Goods," and, with his ghastly promise of speedy cure, sends the young man, who has trusted him, into the arms of the woman who loves him, and wrecks a future with the upas poison of a hopeless future. The commercial press carries the advertisements of these creatures into every home and plays with diabolical skill upon the ignorance of the young and the timid, both selling all that is dearest and sweetest of humanity for the pitiful loot each victim adds as a mite to a mighty monument to Mammon.

It has remained for the masterful hand of the general government to strike a lethal blow at this master crime of commercialism —the traffic in life and honor, and happiness.

Congressman Frank T. O'Hair, of Illinois, the man who defeated Uncle Joe Cannon, has found time, amid all the hurry of the strife over political questions, to introduce a simple bill which will practically shut the doors on this evil, just as some of the more heroic publishers already crippled its prowess.

The bill, which has been referred to the Committee on Interstate and Foreign Commerce, should receive the support of ethical physicians, and of the philanthropic and humanitarian to add the moral support to insure its passage.

The contemplated measure is entitled:

A BILL.

To prevent the use of the United States mail and all common carriers engaged in interstate traffic, in carrying, transporting or conveying any written or printed matter or other device for advertising, selling, delivering or in any way disposing of any false or fraudulent cures for injuries, diseases or physical ailments.

Be it enacted by the Senate and House of Representatives of the United States of America in Congress assembled: That all persons are prohibited from importing into the United States, from any foreign country, or from sending by United States mail or by a common carrier, from any State or territory into any other State or territory, or the District of Columbia into any State or Territory, any newspaper advertisement or any written or printed or partly written and partly printed advertisement, information or device for the selling of any false or fraudulent remedy or cure, or any false or fraudulent remedy or device for the curing of any human disease, injury or ailment.

Section 2. That all persons are prohibited from importing into the United States, and from sending through the United States mail, and from using any common carrier engaged in interstate traffic, in sending, delivering or transporting any false and fraudulent device, instrument or apparatus to be used in attempting falsely and fraudulently to effect any cure for any physical disease, ailment or injury.

Section 3. That no person or corporation owning a newspaper, magazine or other publication, nor any agent or representative thereof, shall publish and send by United States mail or by any common carrier, from one State or Territory, or from the District of Columbia, into another State or Territory, or from any State or Territory into the District of Columbia, any advertisement, publication or other information of, or concerning, any false and fraudulent remedy or cure, or any false or fraudulent remedy or device for the curing of any human disease, injury or ailment; and, for the purpose of enforcing this section of this act, the acts of any corporation, in violating the provisions of this section, shall be taken and considered as the acts of the officers thereof.

Section 4. That any person or persons who knowingly and willfully violate any of the provisions of Sections 1, 2 or 3 of this act shall, for every such offense, be punished by a fine of not more than $5000 or by imprisonment at hard labor for not more than five years, or both, at the discretion of the court.

